# Educational inequalities in health after work exit: the role of work characteristics

**DOI:** 10.1186/s12889-019-7872-0

**Published:** 2019-11-12

**Authors:** Sascha de Breij, Jeevitha Yogachandiran Qvist, Daniel Holman, Jana Mäcken, Jorma Seitsamo, Martijn Huisman, Dorly J. H. Deeg

**Affiliations:** 1 Department of Epidemiology and Biostatistics, Amsterdam Public Health Research Institute, Amsterdam UMC, Vrije Universiteit Amsterdam, De Boelelaan 1089a, 1081 HV Amsterdam, the Netherlands; 2 Centre for Comparative Welfare Studies, Department of Politics and Society, Aalborg University, Fibigerstræde 1 88a, 9220 Aalborg, Denmark; 3 Department of Sociological Studies, University of Sheffield, Sheffield, S10 2TU UK; 4 Institute of Sociology and Social Psychology, University of Cologne, Universitätsstr. 22a, 50937 Cologne, Germany; 5 Department of Work Ability and Working Careers, Finnish Institute of Occupational Health, Topeliuksenkatu 41b, FI-00250 Helsinki, Finland; 6 Department of Sociology, VU University Amsterdam, De Boelelaan, 1081 HV Amsterdam, The Netherlands

**Keywords:** Health inequalities, Post-retirement health, Education, Work characteristics, Mediation analysis, European countries

## Abstract

**Background:**

Educational inequalities in health have been widely reported. A low educational level is associated with more adverse working conditions. Working conditions, in turn, are associated with health and there is evidence that this association remains after work exit. Because many countries are raising the statutory retirement age, lower educated workers have to spend more years working under adverse conditions. Therefore, educational health inequalities may increase in the future. This study examined (1) whether there were educational differences over time in health after work exit and (2) whether work characteristics mediate these educational inequalities in health.

**Methods:**

Data from five prospective cohort studies were used: The Netherlands (Longitudinal Aging Study Amsterdam), Denmark (Danish Longitudinal Study of Aging), England (English Longitudinal Study of Ageing), Germany (German Aging Study), and Finland (Finnish Longitudinal Study on Municipal Employees). In each dataset we used Generalized Estimating Equations to examine the relationship between education and self-rated health after work exit with a maximum follow-up of 15 years and possible mediation of work characteristics, including physical demands, psychosocial demands, autonomy, and variation in activities.

**Results:**

The low educated reported significantly poorer health after work exit than the higher educated. Lower educated workers had a higher risk of high physical demands and a lower risk of high psychosocial demands, high variation in tasks, and high autonomy at work, compared to higher educated workers. These work characteristics were found to be mediators of the relationship between education and health after work exit, consistent across countries.

**Conclusion:**

Educational inequalities in health are still present after work exit. If workers are to spend an extended part of their lives at work due to an increase in the statutory retirement age, these health inequalities may increase. Improving working conditions will likely reduce these inequalities in health.

## Background

Due to the ageing of populations in Europe, many European countries have concerns about securing the financial sustainability of their welfare systems. Thus, pension reforms have been implemented in some countries that raise the statutory pension age and reduce the possibilities of receiving early retirement benefits [1]. The question of whether these reforms might be to the benefit of those most capable to work longer and to the disadvantage of those least capable to work longer, has received too little attention. Yet, studies show large educational inequalities in health [2–5], with some evidence that these inequalities have increased over the last decades [6, 7]. Part of these health inequalities may be attributable to adverse working conditions, which are more prevalent among workers with lower education [8, 9]. Thus, if all workers are to spend an extended part of their lives at work, this may increase health inequalities, even after exit from the workforce. Studies in Western European countries show that social inequalities in self-rated health, depression, disability in daily activities, and mortality indeed persist after retirement [10–15].

With societies being confronted with population ageing, maintaining health in later life is not only desirable from a public health perspective, but it is also becoming increasingly important to prevent health and social care costs from rising. Healthier retirees are also better able than their unhealthy peers to help care for their partners, relatives or grandchildren and to do volunteer work in the community. Therefore, healthy retirees can be an important resource for the economy and for society more broadly [16].

The potential role of work characteristics in explaining health inequalities has received increasing attention during the last decade. The literature suggests that a low educational level is associated with adverse working conditions such as high physical job demands [17, 18] and low control and reward at work [19]. However, some psychosocial job demands such as cognitive demands and time pressure are more common among workers with higher levels of education [9, 20, 21]. Many studies suggest that poor working conditions are associated with poor health [17, 18, 22–25], and there is evidence that this effect remains after work exit [26–30].

Little evidence exists on the role of work characteristics in educational differences in health after work exit. Previous studies that have investigated the association between work characteristics and educational health inequalities have mainly focused on the working age population [31]. Findings from these studies suggest that physical job demands, psychosocial job demands, and psychosocial resources significantly contribute to health inequalities, with these working conditions mediating approximately 25–50% of educational inequalities in health [32–34].

Meanwhile, most studies so far have been cross-sectional. The few longitudinal studies that have investigated the association between work characteristics and health inequalities generally find that working conditions mediate a smaller proportion of the effect of educational level compared to most cross-sectional studies [31]. For example Parker and colleagues [21], who examined health inequalities after retirement, found that working conditions mediated only a small proportion of the association between educational level and self-rated health after retirement. However, the mediating effect in the study depended upon type of working condition as well as the health outcome, e.g., physical working conditions mediated up to 5% of the association between educational level and self-rated health, and 33% of the association between educational level and physical impairments. Psychological working conditions consistently explained very little of the association between educational level and the different measures of health. In contrast, another longitudinal study, by Borg and Kristensen [9], which was conducted among the working age population, found that physical and psychological working conditions together mediate as much as 59% of the association between educational level and self-rated health.

In sum, previous studies suggest that work characteristics partly mediate the association between educational level and health, but evidence remains fragmentary. In particular, there is a need for more longitudinal evidence on the extent to which working conditions mediate the association between educational level and health after work exit. In this cross-national longitudinal study we therefore examine (1) whether educational level is associated with health after work exit, and (2) whether work characteristics mediate the association between educational level and health.

## Methods

EXTEND is a cross-national collaborative project which aims to examine inequalities in relation to extending working lives. We include national datasets from the five countries participating in the EXTEND project: the Netherlands, England, Germany, Denmark, and Finland, to provide a stronger evidence base for examining the role of work characteristics in explaining health inequalities after work exit. The present study adopted a coordinated analysis approach to maximize generalizability across different settings [35].

### Sample

For the Dutch sample, data were used from the Longitudinal Aging Study Amsterdam (LASA). LASA is a nation-wide ongoing longitudinal study in people aged 55+, with follow-ups every three years. The sampling, data collection procedures and non-response have been described in detail elsewhere [36]. Data from the first (respondents aged 55–85 entering the study in 1992–1993), second (new respondents aged 55–65 entering the study in 2002–2003), and third (new respondents aged 55–65 entering the study in 2012–2013) cohorts were pooled for the current study (*n* = 555).

Denmark is represented by the Danish Longitudinal Study of Aging (DLSA), which is merged with Danish register data on labour market exit. DLSA is a longitudinal survey of people aged 52+. The study consists of four consecutive waves with five years between each wave (1997, 2002, 2007 and 2012) and with respondents born in the years between 1920 and 1960. Starting from 2002 a new cohort was added at each new wave. The study is described in more detail elsewhere [37]. In the current study data from all waves (*n* = 1938) were used.

The English data come from the English Longitudinal Study of Ageing (ELSA), which is a study of a large representative sample of men and women aged 50+ living in England. The study began in 2002 and the sample is re-examined every two years [38]. For the current study, data from wave 2 through 7 were used (*n* = 1391), as work characteristics were not measured in wave 1.

The German data come from the German Aging Study (DEAS), a longitudinal survey of the German population aged 40+, the first wave of which was conducted in 1996. Further waves followed in 2002, 2008, 2011 and 2014, with new cohorts added every six years. More detailed information on DEAS can be found elsewhere [39]. Data from four waves since 2002 were used in this study (*n* = 538).

The Finnish data come from the Finnish Longitudinal Study on Municipal Employees (FLAME), collected during 1981–2009. The baseline sample comprised 6257 respondents aged 44–58 and they all had been working at least 5 years in their current occupation. Four waves followed in 1985, 1992, 1997, and 2009. A detailed description of FLAME can be found elsewhere [40]. Altogether 5628 persons were included in this study.

In all datasets respondents were selected who stopped working and participated in at least one wave before and after they exited the workforce. Further inclusion criteria were: at least 50 years old at the last measurement before work exit (T0) and not older than the statutory retirement age at the moment of work exit. The health outcome was measured longitudinally after work exit, because we were interested in both the short-term and long-term health associations. Working conditions were measured at T0. Education and the control variables were not time-varying and were measured at T0.

### Measures

#### Independent variables

##### Educational level

The International Standard Classification of Education 2011 (ISCED 2011) was used to categorize educational level into three groups: low (up to lower secondary education), intermediate (upper secondary education or post-secondary non-tertiary education) and high (short cycle tertiary and higher).

#### Mediators

Because the associations between the continuous measures of the mediators and the outcome were not linear, the mediators were all dichotomized at the median, to maximize comparability between the countries.

##### Physical demands

Data on physical work demands were available in all studies. In the Dutch study, work demands were derived from the general population job exposure matrix (GPJEM) for 55 to 65 year olds [41]. The GPJEM indicates levels of exposure probability of physical and psychosocial demands and psychosocial resources, based on job category. For physical demands, three items were used: use of force, uncomfortable work, and exposure to repetitive movements. Respondents were assigned a low, moderate or high score based on the probability of exposure to these physical demands. A sum score was calculated and dichotomized into low and high exposure to physical demands, based on the median of the sum score.

In the Danish study respondents were asked whether they thought their job requires: too much work using the body, too much lifting and carrying or too many uncomfortable or dislocated positions. Scores were dichotomized into low physical work demands (‘no’ on all three items) and high physical work demands (‘yes’ on at least one item).

In England, participants were asked which of these descriptions, ordered from least to most physically demanding, best describes the work that they do in their main job: (1) sedentary occupation: you spend most of your time sitting (such as in an office), (2) standing occupation: you spend most of your time standing or walking. However the way you spend your time does not require intense physical effort (e.g. shop assistant, hairdresser, security guard, etc.), (3) physical work: this involves some physical effort including handling of heavy objects and use of tools (e.g. plumber, cleaner, nurse, sports instructor, electrician, carpenter, etc.), and (4) heavy manual work: this involves very vigorous physical activity including handling of very heavy objects (e.g. docker, miner, bricklayer, construction worker etc.). Participants were also asked whether their job is physically demanding, with four possible responses from strongly agree to strongly disagree. These two items were summed and dichotomized at the median.

In the German study, physical demands were measured by two questions about strenuous work demands. Respondents were asked to what extent they were stressed by strenuous or repetitive physical activities like carrying heavy objects, standing or sitting for long periods and negative environmental factors such as noise, heat, dust, gases, toxic substances or poor lighting. A sum score was calculated and dichotomized into low and high physical demands, based on the median.

In Finland, physical demands were measured with three items: repetitive work postures, bended, twisted or otherwise difficult work postures, and lifting and holding with hands. Respondents reported if they encountered these demands never, seldom, moderately, often, or very often. The sum score was categorized into low and high physical demands, based on the median.

##### Psychosocial demands

Data on psychosocial work demands were available in all studies. In the Dutch study three items were used to measure psychosocial work demands: time pressure (work at high pace and work under high time pressure), task requirements (work fast, much work, work hard, and hectic work) and cognitive demands (intensive thinking, need to keep focused, and requiring much concentration). Using the aforementioned GPJEM, respondents were assigned a low, moderate or high score based on the probability of exposure to these psychosocial demands. A sum score was calculated and dichotomized into low exposure and high exposure to psychosocial demands, based on the median.

The Danish study used high rate of work, busyness and tight deadlines, lack of influence, and lack of recognition and respect as a measure for psychosocial work demands. Scores were dichotomized into low psychosocial work demands (‘no’ on all four items) and high psychosocial work demands (‘yes’ to at least one item).

The English study used two items to measure psychosocial work demands: working speed (‘Considering the things I have to do at work, I have to work very fast’) and pressure (‘I am under constant pressure due to a heavy workload’). Both items were measured on a 4-point scale (‘strongly agree’ to ‘strongly disagree’). The sum score was dichotomized using the median.

The German study used one question about pressure to complete heavy workloads or meet tight deadlines and nervous tension, which was dichotomized based on the median.

In Finland, psychosocial work demands were measured with three items: being responsible for others, complicated decision making, and urgent decision making and fast solutions. Respondents reported if they encountered these demands never, seldom, moderately, often, or very often. The sum score was categorized into low and high physical demands, based on the median.

##### Variation in tasks

In the Dutch study variation in tasks consisted of three items: variation in work, learn new things, and work requires creativity. It was based on the GPJEM and respondents could be assigned a low, moderate or high score based on the probability of exposure to these resources. The sum score was dichotomized into low and high based on the median.

In Denmark, variation in tasks was measured with the question: ‘Do you think that your work requires too many monotonous and repetitive tasks’? Respondents who answered ‘No’ were categorized as having variations in working activities.

In Finland, variation in activities was measured with one item (‘my work is monotonous and uninteresting’). Respondents replied if this is true at their work not all, little, somewhat, or much. The variable was dichotomized into low and high variation based on the median.

In England and Germany, no measure of variation in tasks was available.

##### Autonomy

In the Dutch sample, autonomy was measured with the following items: decide how to perform the job, the sequence of tasks, work pace, when to take time off, and need to find solutions. It was based on the GPJEM and respondents could be assigned a low, moderate or high score based on the probability of exposure to these resources. The sum score was dichotomized into low and high based on the median.

In the Danish study, autonomy was measured with the following three items: ‘To what extent can you organize your own work, use your qualifications in the right way, use your experience?’*.* All three items were measured on a 3-point scale (‘to a high degree’ to ‘no’). The sum score was dichotomized based on the median.

In the English study, autonomy was measured by two items (‘I feel I have control over what happens in most situations’ and ‘I have very little freedom to decide how I do my work’). Both items were measured on a 4-point scale (‘strongly agree’ to ‘strongly disagree’). The sum score was dichotomized based on the median.

In Germany, no measure of autonomy was available.

In Finland, autonomy was measured with three items: influence your work environment, take part in planning your work, and use your competence and knowledge. The respondents replied according to the options ‘not at all’, ‘little’, ‘somewhat’, or ‘sufficiently’. The sum score was dichotomized based on the median.

#### Dependent variable

##### Self-rated health

Self-rated health (SRH) was chosen as the health measure to distinguish between workers in good and poor health. In the Netherlands, Denmark, England, and Germany, SRH was measured with the question ‘How is your health in general?’ and respondents could answer on a 5-point Likert scale. In the Finnish dataset the question was ‘How do you estimate your health compared to your age mates?’, with response categories ‘much better’, ‘somewhat better’, ‘equal’, ‘somewhat worse’, and ‘much worse’. SRH was recoded so that higher scores reflect better health.

#### Control variables

We controlled for age at work exit, sex, region (not available in the Danish dataset), year, number of working hours, and type of exit. Number of working hours was categorized into four categories representing the most common part-time, full-time and more than full-time working hours in each country. In the Netherlands categories were: 1–15; 16–31; 32–40; ≥41, in Denmark: 1–28; 29–36; 37; ≥38, in England: 1–29; 30–37; 38–44; ≥45, and in Germany: 1–29; 30–39; 40–44; ≥45. Information on the number of working hours was not available in the Finnish dataset. Type of exit was also categorized differently across countries. Categories of work exit in the Netherlands were: regular retirement, early retirement, unemployment, disability, and other; in Denmark: regular retirement, early retirement, and unemployment; in England: (early) retirement, disability, unemployment, and homemaker; in Germany: regular retirement, early retirement, unemployment, and other; and in Finland: regular retirement, disability, and other.

### Missing values

Multiple imputation was used to deal with missing values on the mediator variables, which were assumed to be missing at random. All independent, control and outcome variables were included in the imputation process and the number of imputations was equal to the percentage of incomplete cases in each country [42] (NL: 6.0%; DK: 4.7%; ENG: 17.0%; DE: 20.4%; FI: 21.1%).

### Statistical analysis

**Fig. 1:**
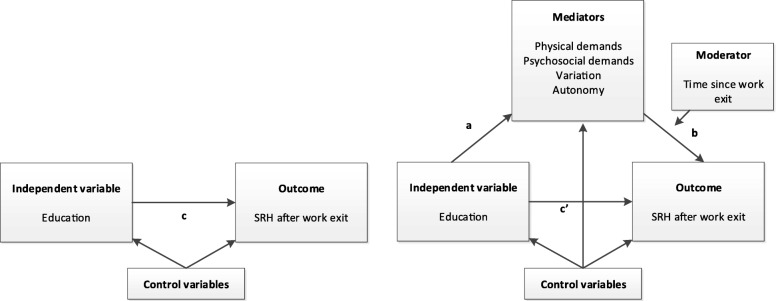
Mediation analyses in the current study

We conducted mediation analyses with single-mediator models. To estimate the c paths (total effect of education on SRH) and b paths (effect of mediators on SRH, controlled for education) we used Generalized Estimating Equations (GEE) with an exchangeable correlation matrix to take into account the clustering in the data due to repeated measures [43]. To calculate the a paths (effect of education on mediators) we used simple logistic regression. The models used to estimate the b paths also yield the estimates for the c’ paths (the direct effect of education on SRH, controlled for the mediator). We used the product-of-coefficients method to calculate the indirect effects [44, 45]. We built separate models for each mediator. Because the effect of work characteristics on health may diminish over time, interaction with time was examined for the b path. In case of a statistically significant (*p* < .10 [46]) interaction, associations were reported for each time point. All models were adjusted for age at work exit, sex, region, year, number of working hours, and type of exit. These analyses were carried out in Stata version 14. The product of a and b represents the indirect, or mediation-, effect [45]. To calculate 95% confidence intervals around these indirect effects, the Monte Carlo method was used [47]. We used the R web utility developed by Selig & Preacher [48], which calculates the 95% confidence intervals around the indirect effects based on the regression coefficients of the a and b paths as well as their standard errors. A visual representation of the models can be found in Fig. 1.

## Results

Characteristics of the samples can be found in Table 1. High physical demands were most prevalent in England (62.3%) and least prevalent in Denmark (32.0%). The highest percentage of workers with high psychosocial demands was found in Germany (70.3%). High variation in tasks was more prevalent in Denmark (77.0%) compared to Finland (46.0%) and the Netherlands (29.2%). High autonomy at work was most common in England (61.6%). The mean age at work exit ranged from 58.6 in Finland to 61.9 in the Netherlands. In the Netherlands and Denmark, early retirement was a common exit route, with a higher prevalence in the higher educated compared to the lower educated. Involuntary work exit, i.e. disability and unemployment routes, was generally more prevalent in the low educated group.

**Table 1:** Characteristics of the samples

	Low Education	Intermediate Education	High Education	Total	% missing before MI^a^
N (%)					
*The Netherlands*	275 (49.5)	131 (23.6)	149 (26.9)	555	6.0
*Denmark*	370 (19.1)	950 (49.0)	618 (31.9)	1938	4.7
*England*	338 (24.3)	724 (52.0)	329 (23.7)	1391	17.0
*Germany*	84 (15.6)	201 (37.4)	253 (47.0)	538	20.4
*Finland*	1608 (31.1)	3250 (62.7)	320 (6.2)	5178	21.1
Male (%)					
*The Netherlands*	52.4	55.7	65.1	56.6	0.0
*Denmark*	38.4	50.0	43.2	45.8	0.0
*England*	53.8	56.4	67.4	58.5	0.0
*Germany*	35.7	50.3	54.9	50.2	0.0
*Finland*	62.6	33.4	49.7	43.5	0.0
Age at work exit, mean (SD)					
*The Netherlands*	61.8 (2.5)	61.9 (2.6)	62.1 (2.3)	61.9 (2.5)	0.0
*Denmark*	60.5 (2.4)	61.0 (2.2)	61.4 (2.2)	61.0 (2.3)	0.0
*England*	60.1 (3.3)	59.8 (3.4)	59.6 (3.0)	59.8 (3.3)	0.0
*Germany*	58.9 (5.1)	60.2 (4.4)	61.2 (4.1)	60.5 (4.4)	0.0
*Finland*	58.5 (3.0)	58.4 (2.7)	60.6 (2.3)	58.6 (2.9)	0.0
High physical demands at T0 (%)					
*The Netherlands*	59.1	49.3	25.5	47.8	5.8
*Denmark*	43.8	37.2	16.8	32.0	0.8
*England*	79.1	62.0	46.9	62.3	16.4
*Germany*	51.2	47.3	46.6	47.6	18.4
*Finland*	59.4	40.8	20.7	45.2	17.4
High psychosocial demands at T0 (%)					
*The Netherlands*	14.8	36.0	81.7	37.8	5.8
*Denmark*	55.7	60.3	65.7	61.1	3.6
*England*	48.3	57.2	68.9	58.0	15.7
*Germany*	58.3	63.2	79.8	70.3	6.9
*Finland*	19.7	47.2	76.3	41.0	17.9
High variation in tasks at T0 (%)					
*The Netherlands*	5.8	27.2	73.9	29.2	5.8
*Denmark*	63.2	74.7	88.9	77.0	0.1
*England*	n/a	n/a	n/a	n/a	n/a
*Germany*	n/a	n/a	n/a	n/a	n/a
*Finland*	31.8	51.5	56.6	46.0	20.0
High autonomy at T0 (%)					
*The Netherlands*	31.7	39.7	42.8	36.5	5.8
*Denmark*	41.6	43.1	47.9	44.3	0.0
*England*	55.4	60.6	69.4	61.6	15.5
*Germany*	n/a	n/a	n/a	n/a	n/a
*Finland*	18.4	42.8	69.6	37.3	18.1
Type of exit (%)					
*The Netherlands*					0.0
Regular retirement	19.6	25.2	18.1	20.5	
Early retirement	39.3	38.2	45.6	40.7	
Disability	9.8	6.9	8.7	8.8	
Unemployment	5.8	9.9	7.4	7.2	
Other	25.5	19.8	20.1	22.7	
*Denmark*					0.0
Regular retirement	7.3	6.8	11.8	8.5	
Early retirement	66.5	72.0	74.3	71.7	
Unemployment	26.2	21.2	13.9	19.8	
*England*					0.0
(Early) retirement	70.3	73.3	84.9	75.5	
Disability	9.3	5.2	1.4	5.2	
Unemployment	12.2	11.2	6.5	10.3	
Homemaker	8.2	10.3	7.2	9.0	
*ȀGermany*					0.0
Regular Retirement	35.7	51.7	60.9	53.5	
Early retirement	19.1	17.9	19.0	18.6	
Unemployment	17.9	13.9	8.3	11.9	
Other	27.4	16.4	11.9	16.0	
*Finland*					0.0
Regular Retirement	41.6	59.1	60.9	53.7	
Disability	50.2	35.4	28.8	39.6	
Other	8.2	5.5	10.3	6.7	
Number of working hours per week at T0 (%)					
*The Netherlands*					1.8
1–15	26.1	21.1	20.8	23.5	
16–31	26.5	20.3	33.6	27.0	
32–40	38.1	40.6	40.3	39.3	
≥ 41	9.3	18.0	5.4	10.3	
*Denmark*					0.3
1–28	16.7	13.9	14.1	14.5	
29–36	18.1	18.2	18.3	18.2	
37	51.1	55.8	47.2	52.2	
≥ 38	14.1	12.1	20.4	15.1	
*England*					3.8
1–15	14.6	9.4	13.0	11.6	
16–31	27.7	28.8	21.5	26.7	
32–40	37.3	40.7	37.3	39.0	
≥ 40	20.4	21.1	28.1	22.7	
*Germany*					7.2
*1–29*	42.9	30.4	24.9	29.7	
*30–39*	19.1	20.9	13.4	17.1	
*40–44*	28.6	28.7	34.0	31.2	
≥ 45	9.5	19.9	27.7	21.9	
*Finland*	n/a	n/a	n/a	n/a	n/a
Self-rated health after work exit, (mean (SD))					
*The Netherlands*					
*T1 (0–3 years after exit)*	3.9 (0.8) *n* = 275	3.8 (0.8) *n* = 131	4.1 (0.8) *n* = 149	3.9 (0.8)	
*T2 (3–6 years after exit)*	3.9 (0.8) *n* = 272	3.8 (0.8) *n* = 130	4.1 (0.7) n = 149	3.9 (0.8)	
*T3 (6–9 years after exit)*	3.8 (0.8) *n* = 209	3.9 (0.6) *n* = 83	4.1 (0.7) *n* = 107	3.9 (0.8)	
*T4 (9–12 years after exit)*	3.7 (0.9) *n* = 196	3.7 (0.9) *n* = 76	4.0 (0.8) *n* = 103	3.8 (0.9)	
*T5 (12–15 years after exit)*	3.8 (0.8) *n* = 163	3.8 (0.8) *n* = 73	4.0 (0.7) *n* = 92	3.8 (0.8)	
*Denmark*					
*T1 (0–5 years after exit)*	3.8 (0.9) *n* = 370	4.0 (0.8) *n* = 950	4.2 (0.8) *n* = 618	4.0 (0.9)	
*T2 (5–10 years after exit)*	3.8 (0.8) *n* = 218	4.0 (0.8) *n* = 505	4.0 (0.8) *n* = 289	4.0 (0.8)	
*T3 (10–15 years after exit)*	3.9 (0.9) *n* = 75	3.9 (0.7) *n* = 143	3.9 (0.8) *n* = 80	3.9 (0.8)	
*England*					
*T1 (0–2 years after exit)*	3.3 (1.1) *n* = 279	3.6 (1.1) *n* = 580	3.8 (0.9) *n* = 291	3.6 (1.1)	
*T2 (2–4 years after exit)*	3.1 (1.1) *n* = 223	3.4 (1.0) *n* = 466	3.8 (1.0) *n* = 245	3.4 (1.0)	
*T3 (4–6 years after exit)*	3.0 (1.0) n = 163	3.4 (1.0) *n* = 352	3.8 (1.0) *n* = 182	3.4 (1.0)	
*T4 (6–8 years after exit)*	3.1 (1.1) *n* = 99	3.3 (1.0) *n* = 216	3.6 (1.0) *n* = 108	3.3 (1.0)	
*T5 (8–10 years after exit)*	3.2 (1.0) *n* = 43	3.3 (1.0) *n* = 79	3.7 (0.9) n = 46	3.4 (1.0)	
*Germany*					
*T1 (0–3 years after exit)*	3.6 (0.9) *n* = 84	3.7 (0.8) *n* = 201	3.7 (0.8) *n* = 253	3.7 (0.8)	
*T2 (3–6 years after exit)*	3.4 (1.0) n = 84	3.6 (0.9) n = 201	3.7 (0.7) n = 253	3.6 (0.8)	
*T3 (6–9 years after exit)*	3.6 (0.8) *n* = 70	3.5 (0.8) *n* = 147	3.6 (0.8) *n* = 197	3.6 (0.8)	
*T4 (9–12 years after exit)*	3.7 (0.7) n = 16	3.5 (0.8) *n* = 57	3.7 (0.8) *n* = 63	3.6 (0.8)	
*Finland*					
*T1 (0–3 years after exit)*	2.7 (1.0) *n* = 1059	3.0 (1.1) *n* = 2617	3.4 (1.0) *n* = 254	2.9 (1.1)	
*T2 (3–6 years after exit)*	2.8 (1.0) *n* = 855	3.2 (1.1) *n* = 2217	3.4 (1.1) *n* = 213	3.1 (1.0)	
*T3 (6–9 years after exit)*	3.0 (1.1) *n* = 519	3.2 (1.0) *n* = 1304	3.4 (1.0) *n* = 94	3.0 (1.1)	

^a^ MI = multiple imputation. Percentages reported in first rows (N) are percentages of incomplete cases

In all countries, those with a low educational level reported a significantly poorer health after work exit than their higher educated peers (Table 2). These associations between educational level and SRH were strongest in England (*b* = −.507). Those with an intermediate educational level also had significantly poorer health after work exit than those with a high educational level. In Germany the difference between the intermediate and the higher educated group was not statistically significant.

**Table 2:** GEE results of the association between education and self-rated health after work exit

	Low Education	Intermediate Education
	c path^a^ (95% CI)	c path^a^ (95% CI)
The Netherlands	−.277 (−.396;-.157)**	−.324 (−.462;-.187)**
Denmark	−.266 (−.364;-.168)**	−.117 (−.193;-.040)**
England	−.507 (−.651;-.362)**	−.241 (−.360;-.121)**
Germany	−.174 (−.345;-.002)*	−.116 (−.235;.004)
Finland	−.461 (−.639;-.282)**	−.220 (−.339;-.101)**

Note: high education is the reference category

* *p* ≤ .05; ** *p* ≤ .01

^a^ B adjusted for age at work exit, sex, year, region, number of working hours, and type of exit

Compared to high educated workers, low educated workers had a statistically significantly higher risk of high physical demands, and a lower risk of high psychosocial demands, high variation in tasks and high autonomy at work (Tables 3, 4, 5, 6, 7, a paths). The b paths represent the associations between the work characteristics and SRH. Interactions with time were included in the models to examine whether the associations were stable over time. If the interactions were statistically significant, coefficients were reported for each time point separately (Tables 3, 4, 5, 6, 7, b paths). In all countries high physical demands were associated with poorer health after work exit. In England, this association was found in the first years after work exit only. In the Netherlands, high psychosocial demands were associated with better health after work exit, but this association was delayed and faded after nine years. In Finland the association was stable over time. In Denmark, England, and Germany high psychosocial demands were associated with poorer health after work exit, although in England and Germany this association faded over time. High variation in tasks was associated with better health after work exit in the Netherlands and Finland with associations remaining up to 15 and 9 years after work exit, respectively, and in Denmark, where the effect was evident in the initial years after exit only. High autonomy at work was also associated with better health after work exit. This association was found in all countries, but in the Netherlands this effect was delayed and faded again after nine years.

**Table 3:** Single-mediator analyses of the effect of education and work characteristics on self-rated health in the Netherlands

	Low Education						Intermediate Education		
	a path^a^ (SE)	b path^a^ (SE)	ab (95% CI)	c’ path^a^ (SE)		a path^a^ (SE)	b path^a^ (SE)	ab (95% CI)	c’ path^a^ (SE)
Physical demands					Physical demands				
*T1*	1.389 (.118)**	−.113 (.056)*	−.157 (−.317;-.006)*	−.245 (.061)**	*T1*	1.141 (.132)**	−.113 (.056)*	−.129 (−.265;-.005)*	−.299 (.070)**
*T2*					*T2*				
*T3*					*T3*				
*T4*					*T4*				
*T5*					*T5*				
Psychosocial demands					Psychosocial demands				
*T1*	−3.430 (.164)**	.008 (.076)	−.027 (−.478;.543)	−.219 (.073)**	*T1*	−2.215 (.150)**	.008 (.076)	−.018 (−.347;.315)	−.284 (.073)**
*T2*		.049 (.075)	−.168 (−.669;.339)		*T2*		.049 (.075)	−.109 (−.436;.219)	
*T3*		.177 (.079)*	−.607 (−1.144;-.070)*		*T3*		.177 (.079)*	−.392 (−.745;-.045)*	
*T4*		.202 (.089)*	−.693 (− 1.298;-.088)*		*T4*		.202 (.089)*	−.447 (−.845;-.057)*	
*T5*		.134 (.086)	−.460 (−1.040;.123)		*T5*		.134 (.086)	−.297 (−.678;.080)	
Variation in tasks					Variation in tasks				
*T1*	−4.255 (.198)**	.153 (.068)*	−.651 (−1.223;-.078)*	−.173 (.075)*	*T1*	−2.264 (.151)**	.153 (.068)*	−.346 (−.656;-.041)*	−.254 (.072)**
*T2*					*T2*				
*T3*					*T3*				
*T4*					*T4*				
*T5*					*T5*				
Autonomy					Autonomy				
*T1*	−.384 (.131)**	.007 (.074)	−.003 (−.065;.058)	−.269 (.062)**	*T1*	−.245 (.143)	.007 (.074)	−.002 (−.039;.034)	−.320 (.070)**
*T2*		.165 (.069)*	−.063 (−.144;-.007)*		*T2*		.165 (.069)*	−.040 (−.080;-.007)*	
*T3*		.158 (.077)*	−.061 (−.145;-.001)*		*T3*		.158 (.077)*	−.039 (−.082;-.002)*	
*T4*		.198 (.089)*	−.076 (−.177;-.006)*		*T4*		.198 (.089)*	−.049 (−.099;-.006)*	
*T5*		.124 (.084)	−.048 (−.132;.015)		*T5*		.124 (.084)	−.030 (−.076;.009)	

Notes: a path = effect of education on the mediators; b path = effect of the mediators on SRH; ab = indirect effect; c’ path = direct effect of education on SRH. If there is no significant interaction with time, coefficients are presented at T1 only

* *p* ≤ .05; ** *p* ≤ .01

^a^ B adjusted for age at work exit, sex, year, region, number of working hours, and type of exit

**Table 4:** Single-mediator analyses of the effect of education and work characteristics on self-rated health in Denmark

	Low Education						Intermediate Education		
	a path^a^ (SE)	b path^a^ (SE)	ab (95% CI)	c’ path^a^ (SE)		a path^a^ (SE)	b path^a^ (SE)	ab (95% CI)	c’ path^a^ (SE)
Physical demands					Physical demands				
*T1*	1.305 (.089)**	−.144 (.038)**	−.188(−.292;-	−.229 (.051)**	*T1*	1.049 (.074)**	−.144 (.038)**	−.151 (−.234;-.071)*	−.088 (.040)*
*T2*			.089)*		*T2*				
*T3*					*T3*				
Psychosocial demands					Psychosocial demands				
*T1*	−.474 (.081)**	−.075 (.035)*	.036 (.003;.073)*	−.274 (.050)**	*T1*	−.252 (.064)**	−.075 (.035)*	.019 (.001;.041)*	−.121(.039)**
*T2*					*T2*				
*T3*					*T3*				
Variation in tasks					Variation in tasks				
*T1*	−1.425 (.098)**	.114 (.046)*	−.162 (−.297;-.032)*	−.248(.051)**	*T1*	−.906 (.087)**	.114 (.046)*	−.103 (−.191;-.022)*	−.108(.039)**
*T2*		−.006(.056)	.009 (−.150;.165)		*T2*		−.006(.056)	.005 (−.093;.108)	
*T3*		−.017(.092)	.024 (−.236; .282)		*T3*		−.017(.092)	.015 (−.147;.181)	
Autonomy					Autonomy				
*T1*	−.154 (.079)*	.083 (.034)*	−.013 (−.175;-.020)*	−.263(.050)**	*T1*	−.113 (.062)*	.083 (.034)*	−.009 (−.027;.002)	−.115(.039)**
*T2*					*T2*				
*T3*					*T3*				

Notes: a path = effect of education on the mediators; b path = effect of the mediators on SRH; ab = indirect effect; c’ path = direct effect of education on SRH. If there is no significant interaction with time, coefficients are presented at T1 only

* *p* ≤ .05; ** *p* ≤ .01

^a^ B adjusted for age at work exit, sex, year, number of working hours, and type of exit

**Table 5:** Single-mediator analyses of the effect of education and work characteristics on self-rated health in England

	Low Education						Intermediate Education		
	a path^a^ (SE)	b path^a^ (SE)	ab (95% CI)	c’ path^a^ (SE)		a path^a^ (SE)	b path^a^ (SE)	ab (95% CI)	c’ path^a^ (SE)
Physical demands					Physical demands				
*T1*	1.412 (.117)**	−.139 (.068)*	−.196 (−.394;-.010)*	−.483 (.076)**	*T1*	0.552 (.076)**	−.139 (.068)*	−.077 (−.159;-.004)*	−.234 (.061)**
*T2*		−.060 (.068)	−.085 (−.278;.102)		*T2*		−.060 (.068)	−.033 (−.111;.040)	
*T3*		.018 (.079)	.025 (−.196;.245)		*T3*		.018 (.079)	.010 (−.076;.097)	
*T4*		.068 (.094)	.096 (−.165;.359)		*T4*		.068 (.094)	.038 (−.063;.143)	
*T5*		.172 (.130)	.243 (−.110;.610)		*T5*		.172 (.130)	.095 (−.043;.244)	
Psychosocial demands					Psychosocial demands				
*T1*	−1.058 (.105)**	−.154 (.072)*	.163 (.015;.322)*	−.523 (.075)**	*T1*	−.662 (.089)**	−.154 (.072)*	.102 (.009;.206)*	−.254 (.062)**
*T2*		−.104 (.070)	.110 (−.033;.262)		*T2*		−.104 (.070)	.069 (−.021;.167)	
*T3*		−.030 (.077)	.032 (−.127;.196)		*T3*		−.030 (.077)	.020 (−.081;.123)	
*T4*		−.012 (.092)	.013 (−.178;.208)		*T4*		−.012 (.092)	.008 (−.114;.128)	
*T5*		−.193 (.126)	.204 (−.059;.474)		*T5*		−.193 (.126)	.128 (−.036;.301)	
Autonomy					Autonomy				
*T1*	−.628 (.118)**	.200 (.060)**	−.126 (−.222;-.046)*	−.479 (.074)**	*T1*	−.366 (.100)**	.200 (.060)**	−.073 (−.140;-.023)*	−.223 (.060)**
*T2*					*T2*				
*T3*					*T3*				
*T4*					*T4*				
*T5*					*T5*				

Notes: a path = effect of education on the mediators; b path = effect of the mediators on SRH; ab = indirect effect; c’ path = direct effect of education on SRH. If there is no significant interaction with time, coefficients are presented at T1 only

* *p* ≤ .05; ** *p* ≤ .01

^a^ B adjusted for age at work exit, sex, year, region, number of working hours, and type of exit

**Table 6:** Single-mediator analyses of the effect of education and work characteristics on self-rated health in Germany

	Low Education						Intermediate Education		
	a path^a^ (SE)	b path^a^ (SE)	ab (95% CI)	c’ path^a^ (SE)		a path^a^ (SE)	b path^a^ (SE)	ab (95% CI)	c’ path^a^ (SE)
Physical demands					Physical demands				
*T1*	.266 (.173)	−.266 (.061)**	−.071 (−.178;.020)	−.156 (.086)	*T1*	.151 (.131)	−.266 (.061)**	−.040 (−.163;.053)	−.105 (.060)
*T2*					*T2*				
*T3*					*T3*				
*T4*					*T4*				
Psychosocial demands					Psychosocial demands				
*T1*	−.618 (.210)**	−.261 (.078)**	.161 (.038;.331)*	−.189 (.088)**	*T1*	−.820 (.129)**	−.261 (.078)**	.214 (.083;.370)*	−.139 (.061)*
*T2*		−.128 (.085)	.079 (−.024;.218)		*T2*		−.128 (.085)	.105 (−.028;.253)	
*T3*		−.150 (.084)	.092 (−.010;.238)		*T3*		−.150 (.084)	.123 (−.012;.273)	
*T4*		.029 (.137)	−.018 (−.210;.160)		*T4*		.029 (.137)	−.024 (−.252;.204)	

Notes: a path = effect of education on the mediators; b path = effect of the mediators on SRH; ab = indirect effect; c’ path = direct effect of education on SRH. If there is no significant interaction with time, coefficients are presented at T1 only

* *p* ≤ .05; ** *p* ≤ .01

^a^ B adjusted for age at work exit, sex, year, region, number of working hours, and type of exit

**Table 7:** Single-mediator analyses of the effect of education and work characteristics on self-rated health in Finland

	Low Education						Intermediate Education		
	a path^a^ (SE)	b path^a^ (SE)	ab (95% CI)	c’ path^a^ (SE)		a path^a^ (SE)	b path^a^ (SE)	ab (95% CI)	c’ path^a^ (SE)
Physical demands					Physical demands				
*T1*	1.852 (.159)**	−.107 (.034)**	−.198 (−.506;-.110)*	−.420 (.089)**	*T1*	.775 (.152)**	−.107 (.034) **	−.083 (−.152;-.027)*	−.205 (.059)**
*T2*					*T2*				
*T3*					*T3*				
*T4*					*T4*				
Psychosocial demands					Psychosocial demands				
*T1*	−2.613 (.161)**	.072 (.036)	−.188 (−.337;-.004)*	−.419 (.092)**	*T1*	−1.285 (.152)**	.072 (.036)	−.093 (−.191;-.001)*	−.199 (.063)**
*T2*					*T2*				
*T3*					*T3*				
*T4*					*T4*				
Variation in tasks					Variation in tasks				
*T1*	−0.962 (.134)**	.077 (.037)*	−.074 (−.151;-.004)*	−.437(.081)**	*T1*	−0.320 (.130)*	.077 (.037)*	−.025 (−.062;.0001)	−.212 (.057)**
*T2*					*T2*				
*T3*					*T3*				
*T4*					*T4*				
Autonomy					Autonomy				
*T1*	− 2.075(.141)**	.113 (.032)**	−.234 (−.370;-.105)*	−.409(.090)**	*T1*	−0.957 (.127)**	.113 (.032)**	−.108 (−.179;-.047)*	−.193 (.061)**
*T2*					*T2*				
*T3*					*T3*				
*T4*					*T4*				

Notes: a path = effect of education on the mediators; b path = effect of the mediators on SRH; ab = indirect effect; c’ path = direct effect of education on SRH. If there is no significant interaction with time, coefficients are presented at T1 only

* *p* ≤ .05; ** *p* ≤ .01

^a^ B adjusted for age at work exit, sex, year, region, and type of exit

Results suggested that all work characteristics were mediators in the association between educational level and health after work exit (Tables 3, 4, 5, 6, 7, ab). However, even after including these mediators in the models, an association of educational level with health after work exit remained (Tables 3, 4, 5, 6, 7, c’ paths).

## Discussion

The aim of our study was to examine whether educational level is associated with health after work exit in five Northern and Western European countries, and whether work characteristics mediate the association between educational level and health after work exit.

Consistent with earlier studies reporting educational health inequalities after work exit [10, 12–15], we found that the lower educated reported significantly poorer health than the higher educated. The association between educational level and health after work exit differed by country. We found the largest associations between educational level and health in England and Finland, and smaller, but still statistically significant associations in the Netherlands, Denmark, and Germany.

Next, we examined the associations between educational level and work characteristics, and the associations between work characteristics and health after work exit (while controlling for educational level). Consistent with the empirical literature [8, 9], we found that lower educated workers had a higher risk of high physical demands, and a lower risk of high psychosocial demands, high variation in tasks and high autonomy at work, compared to higher educated workers. We also found that work characteristics were associated with health after work exit, sometimes even up to 12–15 years. The duration of these associations differed by country and by work characteristic. The negative association between physical demands and health was apparent even years after exiting the work force in all countries except for England, where this association diminished after the initial years after exit. The positive effects of psychosocial resources at work, i.e. variation in tasks and autonomy, generally were also still present many years after work exit. Results on psychosocial demands were mixed. In the Netherlands and Finland psychosocial demands were associated with better health after work exit, whereas psychosocial demands were associated with poorer health in England, Denmark, and Germany. These divergent findings may be due to differences in the constructs measured across the countries. In the Netherlands and Finland, psychosocial demands were mainly operationalized as cognitive demands e.g. having to make complicated decisions and doing tasks that require a lot of concentration. In the other countries, psychosocial demands consisted mainly of items measuring time pressure and heavy work load. This suggests that the cognitive demands can be seen more as a positive challenge at work, which is likely beneficial for your health, whereas demands such as working under time pressure are associated with poorer health. Therefore, the mediated and direct effect had opposite signs in England, Denmark, and Germany, which led to a suppression effect for psychosocial demands in these countries, i.e. the association between educational level and health was larger after including these suppressors in the models [49]. Results on the duration of the effect of psychosocial demands on health after work exit were mixed, with longer lasting effects in the Netherlands and Denmark, and more short-term effects in England and Germany.

We found that physical demands, psychosocial demands, variation in tasks and autonomy at work all partially mediated the association between educational level and self-rated health after work exit. Although there were some country differences, these mediating effects were generally observed in all five countries. However, after including these mediators into the statistical models, substantial associations between educational level and health after work exit remained. Parker et al. concluded in their longitudinal study on post-retirement health that physical demands partially explained the association between education and physical impairment, but not between education and self-rated health. They did not find evidence for a mediating effect of psychosocial demands [21]. These differences in findings may be due to different measures and different methods to analyze the mediation effects. Parker et al. dichotomized educational level into lower education (mandatory education only) and higher education (more than mandatory education), while we used the ISCED categories low, intermediate and high educational level. Physical working conditions (‘In your work situation, are you exposed to gas, dust, smoke, noise, and/or heavy lifting?’) and psychological working conditions (‘Is your work mentally taxing, stressful, repetitious, monotonous, or mentally exhausting?’) were each measured with one item in their study. While Parker et al. only examined changes in coefficients, we modeled each path and therefore gained more insight in the underlying mediation mechanisms. Also, we made full use of our longitudinal data by including interactions with time to examine changes over time in the mediation effects. Furthermore, we not only included physical and psychosocial work demands, but also included psychosocial resources: variation in tasks and autonomy, which were also found to be mediators.

In view of the necessity to spend more years working due to an increase in the statutory retirement age, our results indicate that it is important to adapt working conditions to improve health and reduce health inequalities. Our study provides evidence to suggest that physical demands, psychosocial demands, variation in tasks, and autonomy are associated with health and that they partly mediate the association between education and health. Even years after work exit, associations between work characteristics and health still exist. Work place interventions improving working conditions, may improve the health of all retirees as well as decrease educational inequalities therein. Participatory ergonomics interventions, in which workers are actively involved in developing and implementing changes in the workplace, may be promising to reduce physical demands at work [50]. Measures to enhance variation and autonomy could be job rotation, which involves moving employees from job to job at regular intervals; job enlargement, which refers to expanding the tasks to add more variety; and job enrichment, which gives workers more responsibility and control over how they perform their own tasks. Because working conditions explain only part of the educational inequalities in health, inequalities are likely to be reduced but not dissolved when improving these conditions. Therefore, health interventions, especially those aimed at the lower educated, should also be implemented to promote health and reduce health inequalities. It has also been argued that education itself should be considered as a domain of public health [51, 52].

The present study has some limitations*.* First, in all countries only characteristics of the last held job were used. However, it is possible that those with worse health already changed jobs to accommodate their health better, which may have attenuated our results [53]. Therefore, our results should be replicated by studies investigating the role of characteristics of the longest held job. Second, not all work characteristics were measured in all countries. For instance, information about variation in tasks and autonomy at work was not available for Germany. The mediating role of psychosocial resources, i.e. variation in tasks and autonomy at work, can therefore not be generalized to the German context.

Third, we included only SRH as our health outcome because it was the only health measure available in all datasets. SRH can be used as a global measure of health in the general population [54]. It has previously been associated with other health measures, e.g. depression [55], inflammation [56], functional limitations [57], and mortality [58]. However, studies show that there may be educational differences in the relation between objective health and SRH, and thus results may be either over- or underestimating educational health inequalities [59]. Therefore, results should be interpreted with caution when using SRH as a proxy for objective health. In our study, however, SRH is seen as a global measure of people’s perception of their health, and we refrained from making claims about associations of education and job characteristics with specific objective health outcomes [60, 61]. Furthermore, because of relatively small sample sizes in some of the countries, we did not examine multiple mediators in one model. The next step would be to also examine these parallel mediation models, because the mediators are likely to be interdependent and may be together part of a causal mechanism that is more complex than what we could test in our study. Finally, differences between countries in effect sizes may be due to factors on the country level we did not control for in our study, e.g. generosity of benefits.

This study also has important strengths. Most research has focused on the working population and used cross-sectional data. We included five longitudinal datasets, following respondents well into retirement and included five of the highest income countries in Europe with different welfare regimes. A further strength is that the effects found were consistent across countries, despite potential differences in how they were operationalized. The exception to this was psychosocial demands. Further work is needed given disparate measures across national datasets.

## Conclusion

Our longitudinal, cross-national study demonstrated educational inequalities in self-rated health after work exit in the Netherlands, Denmark, England, Germany, and Finland. These educational inequalities were partially mediated by physical demands, psychosocial demands, variation in tasks and autonomy at work. The associations between these work characteristics and health sometimes lasted up to 12–15 years after having exited the work force. Thus, if workers are to spend an extended part of their lives at work, health inequalities may increase, not only in recent retirees, but also years after work exit. Improving these working conditions will likely reduce, but not dissolve, educational health inequalities after work exit. In addition, health interventions and promotion targeting the lower educated retirees, especially those who experienced unfavorable work demands, may prove to be important in improving health and diminishing health inequalities in older adults.

## Data Availability

Data from the Longitudinal Aging Study Amsterdam (LASA; www.lasa-vu.nl) are available for use for specific research questions provided that an agreement is made up. Data from the English Longitudinal Study of Ageing (ELSA) are available from the UK Data Archive (http://data-archive.ac.uk/). Data from the Danish Longitudinal Study of Ageing (DLSA) are available from the Centre for survey and Survey/Register data (CSSR) (http://cssr.surveybank.aau.dk/webview/). Data from the German Aging Study (DEAS) are available from the German Centre of Gerontology (https://www.dza.de/en/fdz/german-ageing-survey/access-to-deas-data.html). Data from the Finnish Longitudinal Study of Ageing Workers (FLAME) are available for use for specific research questions provided that an agreement is made up.

## Abbreviations

B: Unstandardized coefficient
CI: Confidence interval
DEAS: German Aging Study
DLSA: Danish Longitudinal Study of Aging
ELSA: English Longitudinal Study of Ageing
FLAME: Finnish Longitudinal Study on Municipal Employees
GEE: Generalized Estimating Equations
GPJEM: General Population Job Exposure Matrix
ISCED: International Standard Classification of Education
LASA: Longitudinal Aging Study Amsterdam
MI: multiple imputation
SD: standard deviation
SE: standard error
SRH: self-rated health
